# Changes in ocular biometric measurements after vitrectomy with silicone oil tamponade for rhegmatogenous retinal detachment repair

**DOI:** 10.1186/s12886-020-01627-2

**Published:** 2020-09-02

**Authors:** Rui Liu, Qingchen Li

**Affiliations:** 1 Department of Ophthalmology, Shanghai Jing’an District Shibei Hospital, Shanghai, 200443 China; 2 Department of Ophthalmology and Vision Science, Eye and ENT Hospital, Fudan University, 83# Fenyang Road, Xuhui District, Shanghai, 200031 People’s Republic of China; 3 Key Laboratory of Myopia of State Health Ministry, and Key Laboratory of Visual Impairment and Restoration of Shanghai, Shanghai, 200031 China; 4 Laboratory of Myopia, Chinese Academy of Medical Sciences, Shanghai, 200031 People’s Republic of China

**Keywords:** Rhegmatogenous retinal detachment, Myopic shift, Vitrectomy, Silicone oil tamponade, Ocular biometric measurement

## Abstract

**Background:**

To observe the changes in ocular biometric measurements after vitrectomy with silicone oil tamponade for rhegmatogenous retinal detachment (RRD) repair.

**Methods:**

Sixty-three phakic, macula-off RRD eyes underwent vitrectomy with silicone oil tamponade but not lens extraction were included in this retrospective study. Measurements of axial length (AL), anterior chamber depth (ACD), lens thickness (LT) using the new Zeiss IOLMaster 700 and derivative lens position (LP), relative lens position (RLP) and lens-retina distance (LRD) were compared and analyzed between preoperative and postoperative in phakic, macula-off eyes with RRD.

**Results:**

Preoperative AL, ACD, LT, LP, RLP and LRD were 24.94 ± 1.82 mm, 3.45 ± 0.42 mm, 4.34 ± 0.16 mm, 5.55 ± 0.41 mm, 0.22 ± 0.01 and 19.52 ± 1.82 mm. After a mean 4.85-month duration of silicone oil tamponade, postoperative AL, ACD, LT, LP, RLP and LRD were 25.42 ± 2.20 mm, 3.30 ± 0.41 mm, 4.43 ± 0.21 mm, 5.46 ± 0.40 mm, 0.22 ± 0.02 and 20.17 ± 2.36 mm, respectively. The differences in all measurements are significant (all *P* < 0.05). Preoperative AL and LRD are positively while RLP is negatively correlated with change in LRD. Change in AL but not in LT or LP is correlated with change in LRD. Biometric measurements except LT between preoperative and postoperative were in close agreement.

**Conclusion:**

The underestimation of AL and anterior shifting of lens in phakic, macula-off eyes with RRD after vitrectomy with silicone oil tamponade.

## Background

Rhegmatogenous retinal detachment (RRD) is a disease that occurs when liquefied vitreous humor flows into the potential space between the neurosensory retina and the underlying retinal pigment epithelium, resulting in their separation [1, 2]. Due to the complications of coexisting cataract and nuclear sclerotic cataract, patients with RRD undergoing vitrectomy often require subsequent cataract extraction for visual rehabilitation [3]. Combined phacoemulsification, intraocular lens implantation and pars plana vitrectomy, known as phacovitrectomy, has become a widely performed surgical procedure in patients aged 50 and older with vitreoretinal pathologies [4] that reduces costs and offers faster visual rehabilitation by avoiding the need for additional surgery and allowing a single recovery period [5]. However, phacovitrectomy may induce significant postoperative myopic shift [2, 6], particularly in macula-off RRD cases [5, 7]. The reason for the myopic shift after phacovitrectomy for RRD repair is controversial. An underestimation of axial length (AL) preoperatively and postoperatively by acoustic biometry (A-scan ultrasound) represents a common source of deviation in intraocular lens power calculation [8], which seems to be insignificant using optical methods (IOLMaster) [9]. The relative lens position (RLP) is defined as the lens location relative to the axial length (AL) of the globe [10], but to our knowledge, whether changes in lens position (LP) and RLP occur after phacovitrectomy, which might be a potential explanation for refractive error, are unclear. A new noncontact swept-source optical coherence tomography-based IOLMaster 700 allows accurate and convenient measurement of AL, anterior chamber depth (ACD) and lens thickness (LT) in subjects with both clear crystalline lenses [11] and cataracts [12]. Using this technique, we conducted a retrospective, self-control study to examine changes in ocular biometric measurements of phakic, macula-off eyes undergoing vitrectomy with silicone oil tamponade for RRD repair.

## Methods

### Participants

This retrospective, self-control study was conducted in the Eye and ENT Hospital, Fudan University, between September 2017 and April 2019 with ethical approval granted by the associated ethics committee, adhering to the tenets of the Declaration of Helsinki and with written informed consent from all participants. Phakic, macula-off RRD eyes that underwent vitrectomy with silicone oil tamponade but not lens extraction were recruited, while eyes with scleral buckling surgery, recurrent retinal detachment or any other ocular problems affecting biometric measurements, such as corneal scar and lens dislocation, were excluded [13].

### Surgical procedure

A standard 23-gauge pars plana vitrectomy was performed using the CONSTELLATION® Vision 106 System (Alcon Laboratories, Inc.). The RESIGHT™ Fundus Viewing System (Carl Zeiss Meditec Inc.) was used during vitrectomy. Core vitrectomy, mid-peripheral vitrectomy, and vitreous base shaving under scleral depression was performed to remove the vitreous as similar studies described [4]. According to the extent of retinal detachment, Perfluorocarbon liquid (Perfluoron; Alcon Laboratories, Inc.) might be used in some cases. Endolaser photocoagulation was performed around the retinal tears, and fluid–air exchange was performed before silicone oil injection (Oxane 5700 cSt; Bausch & Lomb Inc., Waterford, Ireland).

### Data collection

Preoperative AL, ACD and LT of RRD eyes were measured using an IOLMaster 700 (Carl Zeiss Meditec Ltd., Jena, Germany) as formerly described [14, 15]. According to Kunavisarut [13] and El-Khayat [16], only measurements from an IOLMaster with a signal-to-noise ratio (SNR) value greater than 2 were included. Postoperative measurements were also obtained using the IOLMaster 700 with silicone oil-filled phakic eyes program before silicone oil removal. Mathematical LP and RL*P* values were calculated according to Nongpiur’s formula as follows [17]:

$$ \mathrm{LP}=\mathrm{ACD}+\frac{1}{2} LT $$

and

$$ \mathrm{RLP}=\frac{\mathrm{LP}}{AL.} $$

The smaller the RLP value, the more relatively anterior the position of the lens in the globe. We set a new parameter, “Lens-Retina Distance (LRD) = AL − LP ”, to describe the axial length from the center of the lens to the retina, calculating the changes in all ocular biometric measurements (△AL, △ACD, △LT, △LP, △RLP and △LRD) as “postoperative - preoperative”.

### Statistical analysis

Continuous variables are expressed as the mean ± standard deviation. Statistical analyses were performed using independent samples t-test and paired samples t-test with SPSS Statistics 26.0 for Windows (SPSS Inc., Chicago, IL, USA). Univariate linear regression was performed to show correlations between patient characteristics and changes in measurements. Bland–Altman plots were performed using MedCalc Statistical Software (version 15.0; MedCalc Software, Inc., Mariakerke, Belgium) to assess the agreement of biometric measurements between preoperative and postoperative data. Statistical significance was set at *P* < 0.05.

## Results

**Fig. 1:**
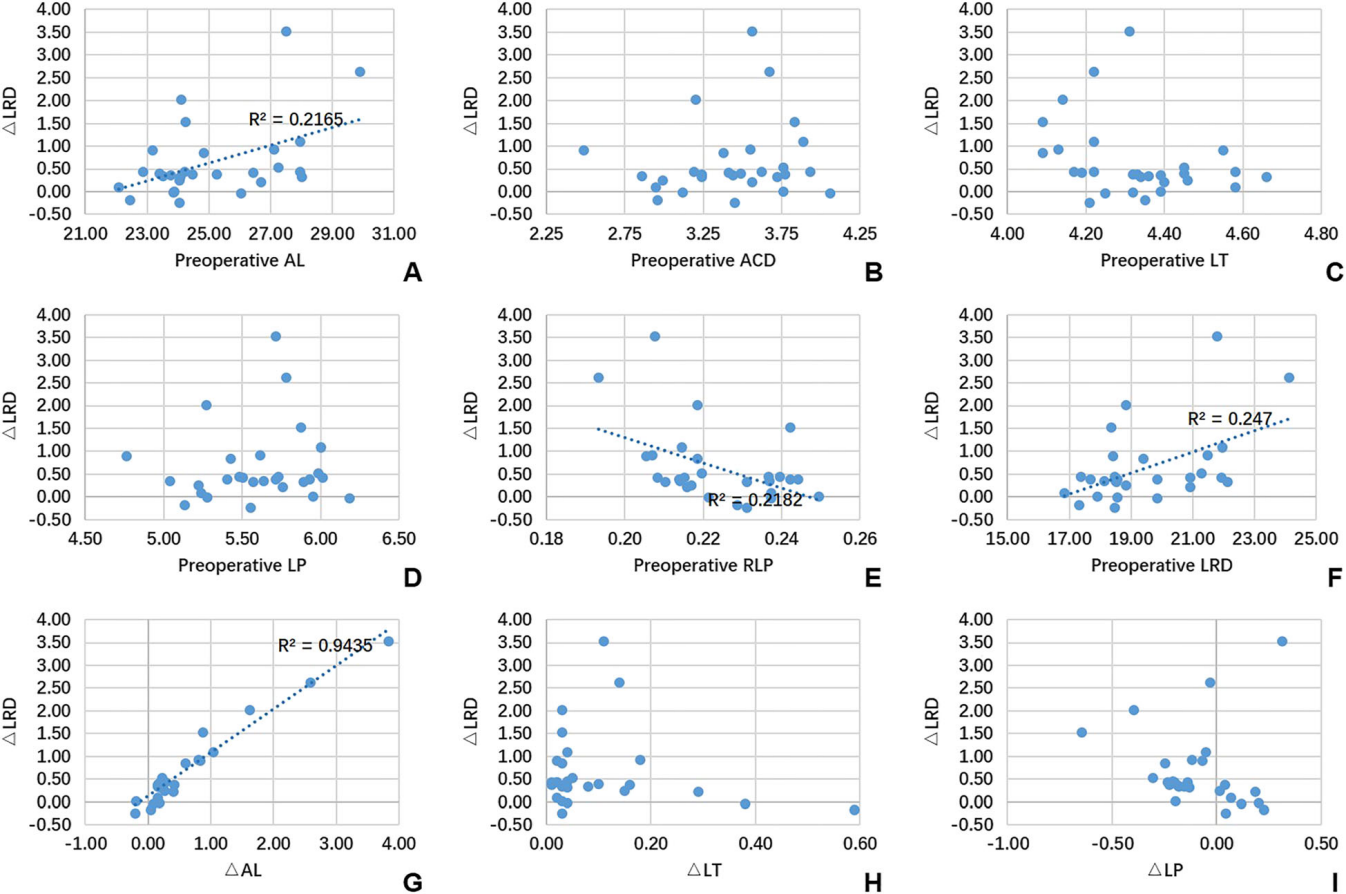
Univariate linear regressions between △LRD and biometric measurements. Preoperative AL and LRD are positively correlated with △LRD, while RLP is negatively correlated with △LRD (all *P* < 0.05). None of preoperative ACD, LT or LP is correlated with △LRD (all *P* > 0.05), and △AL (P < 0.05) but not △LT or △LP (P > 0.05) is correlated with △LRD

**Fig. 2:**
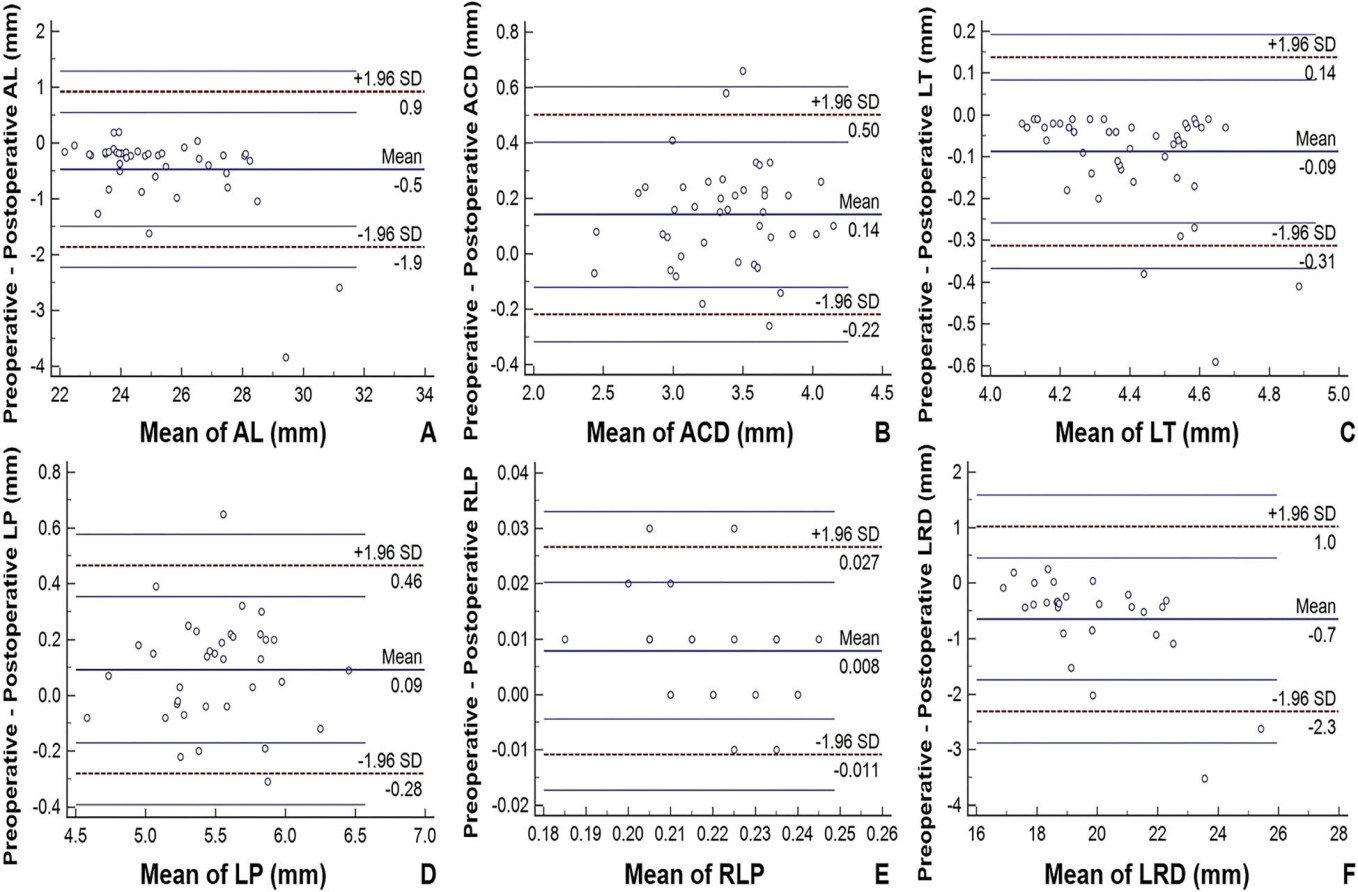
Bland-Altman plots of AL, ACD, LT, LP, RLP and LRD between preoperative and postoperative data. AL, ACD, LP, RLP and LRD (more than 95% cases were included in 95% limits of agreement) but not LT (50/53, 94.3% cases were included in 95% limits of agreement) were in close agreement

Data from 63 eyes of 63 patients (34 males and 29 females) with an age range from 28 to 75 years (mean 51.02 ± 9.60 years) were included in the study. Examination failure rates of preoperative AL, ACD and LT were 28.6, 22.2 and 14.3% and for postoperative data were 0, 15.9 and 3.2%, respectively. The mean duration of silicone oil tamponade was 4.85 ± 1.85 months. Independent samples t-test demonstrated there was no significant difference in either preoperative or postoperative AL, ACD, LT, LP, RLP, LRD, or in the duration of silicone oil tamponade between genders (all *P* > 0.05). Table 1 summarizes the data measured in RRD eyes and silicone oil-filled eyes. Preoperative AL, ACD, LT, LP, RLP and LRD were 24.94 ± 1.82 mm, 3.45 ± 0.42 mm, 4.34 ± 0.16 mm, 5.55 ± 0.41 mm, 0.22 ± 0.01 and 19.52 ± 1.82 mm, respectively, and postoperative AL, ACD, LT, LP, RLP and LRD were 25.42 ± 2.20 mm, 3.30 ± 0.41 mm, 4.43 ± 0.21 mm, 5.46 ± 0.40 mm, 0.22 ± 0.02 and 20.17 ± 2.36 mm, respectively. Differences in AL, ACD, LT, LP, RLP and LRD between preoperative and postoperative measurements were all statistically significant (all *P* < 0.05). Classifying patients based on preoperative data into a “highly myopic group” as “AL > =26.00 mm” and a “non-highly myopic group” as “AL < 26.00 mm” followed by independent samples t-test showed no significant difference in △AL, △ACD, △LT, △LP, △RLP, or △LRD between either the myopic groups or genders (all *P* > 0.05). Univariate linear regression revealed that age is positively correlated with △RLP and negatively correlated with △LRD. Furthermore, the duration of silicone oil tamponade is positively correlated with △LT (Table 2). Scatter diagrams presented in Fig. 1 demonstrate the linear regressions between preoperative biometric data and △LRD. Preoperative AL and LRD are positively, while RLP is negatively, correlated with △LRD. None of the ACD, LT or LP preoperative measurements were correlated with △LRD. These findings indicate that △AL, but not △LT or △LP, is significantly correlated with △LRD. Bland–Altman plots indicate that the agreements of biometric measurements between preoperative and postoperative data were acceptable in AL, ACD, LP, RLP and LRD (more than 95% of cases were included in 95% limits of agreement), with the exception of LT (50/53, 94.3% of cases were included in 95% limits of agreement), as shown in Fig. 2.

**Table 1:** The comparison of measurements between preoperative and postoperative

	n (pairs)	Preoperative		Postoperative		Difference (post-pre)		Paired samples t-test	
		Mean	SD	Mean	SD	Mean	95% CI	t value	P value^*^
AL (mm)	45	24.94	1.82	25.42	2.20	0.48	0.26, 0.69	−4.493	**< 0.001**
ACD (mm)	42	3.45	0.42	3.30	0.41	− 0.14	−0.20, − 0.08	4.974	**< 0.001**
LT (mm)	53	4.34	0.16	4.43	0.21	0.09	0.06, 0.12	−5.564	**< 0.001**
LP (mm)	36	5.55	0.41	5.46	0.40	−0.09	−0.16, − 0.03	2.862	0.007
RLP	28	0.22	0.01	0.22	0.02	−0.01	−0.01, 0	4.458	**< 0.001**
LRD (mm)	28	19.52	1.82	20.17	2.36	0.65	0.32, 0.98	−4.059	**< 0.001**

^*^P values less than 0.05 are represented in bold

*AL* axial length, *ACD* anterior chamber depth, *LT* lens thickness, *LP* lens position, *RLP* relative lens position, *LRD* lens-retina distance

**Table 2:** Univariate linear regression between patient characteristics and changes of biometric measurements

Predictors^†^	n (pairs)	Age (years)			Duration of silicone oil tamponade (months)		
		β	P value^*^	r^2^	β	P value^*^	r^2^
△AL (mm)	45	−0.291	0.052	0.085	−0.078	0.610	0.006
△ACD (mm)	42	0.260	0.097	0.067	0.001	0.997	< 0.001
△LT (mm)	53	−0.120	0.391	0.14	0.817	**< 0.001**	0.668
△LP (mm)	36	0.249	0.144	0.062	0.234	0.170	0.055
△RLP	28	0.380	**0.046**	0.144	0.283	0.144	0.080
△LRD (mm)	28	−0.439	**0.019**	0.193	−0.148	0.453	0.022

^†^△ = “postoperative - preoperative”

^*^P values less than 0.05 are represented in bold

*AL* axial length, *ACD* anterior chamber depth, *LT* lens thickness, *LP* lens position, *RLP* relative lens position, *LRD* lens-retina distance

## Discussion

In the present study, AL, ACD, and LT, as well as derivative LP, RLP and LRD, were measured in phakic, macula-off eyes with RRD and compared to those recorded after a successful vitrectomy and silicone oil tamponade. Results revealed that, after a mean 4.85-month duration of silicone oil tamponade, AL was approximately 0.48 mm longer than in RRD status eyes, ACD was 0.14 mm shallower, and LT was 0.09 mm thicker, causing an LP anterior move of 0.09 mm, an RLP decrease of 0.01 and an LRD increase of 0.65 mm. Therefore, a postoperative myopic shift occurs if phacovitrectomy was performed according to the preoperative data, although this effect might be neutralized by a hyperopic shift during the period of silicone oil tamponade [18–20]. Previous studies have suggested that myopic shift results from underestimation of AL using A-scan ultrasonography because it measures the distance from the cornea to the inner limiting membrane as AL [6]. IOLMaster is more accurate with less deviation in its predictive postoperative refractive error than A-scan ultrasound, as it measures from the front of cornea to the retinal pigment epithelium [13]. However, in some macula-off RRD eyes, a similar strong interference from interfaces in the detached retina may provide a good signal-to-noise ratio measurement, even though the result is incorrect [21], partly explaining the conflicting findings of studies measuring preoperative and postoperative AL using IOLMaster. For instance, Pongsachareonnont et al. found that underestimation of AL in macular involvement eyes with RRD was 0.59 ± 0.90 mm [22], while Kim et al. thought this underestimation was associated with macular retinal detachment height [7]. Furthermore, Shiraki et al. reported that AL measurement was not associated with postoperative myopic shift and considered AL to be correctly underestimated even in eyes with macula-off RRD [1]. Using the IOLMaster 700, which provides a 44-mm scan depth, captures 2000 A-scans per second for the full-eye length tomogram acquisition and shows anatomical details of a longitudinal cut through the entire eye [15], we observed an underestimation of AL in eyes with macula-off RRD. Furthermore, ACD of RRD eyes was also decreased by an average of 0.14 mm after vitrectomy, consistent with previous studies [23]. Huang et al. attributed this phenomenon to abnormally low intraocular pressure prior to surgery, which results in falsely high measurements of ACD, postoperatively recovered intraocular pressure, and an operative wound, which stimulates contraction of the ciliary body muscle to induce increased lens convexity and LT [9]. In addition, the facedown position and silicone oil tamponade may also shift the lens-iris diaphragm forward.

Theoretically, an underestimation of AL and an anterior shifting of the lens location both contribute equally to postoperative refractive error. We established the parameter LRD as AL minus LP to combine both factors. In our study, △LRD was only positively correlated with baseline AL and LRD but not preoperative ACD, LT or LP. Furthermore, underestimation of AL, rather than the thickening of LT or anterior shifting of the lens, is correlated with △LRD, suggesting that underestimating AL is the major cause of postoperative myopic shift. Kang et al. reported a postoperative myopic shift of 0.41 ± 0.67 diopters in patients with macula-sparing RRD following phacovitrectomy compared to the predicted value, considering this shift to be primarily caused by factors affecting the intraocular lens position, such as preoperative ACD and LT, rather than a change in AL [2]. The present study confirmed a 0.09-mm anterior shift of the lens in silicone oil-filled eyes after RRD repair. According to Sun et al., a measurement error of 100 μm results in a postoperative refractive error of 0.25 diopters following the SRK formula [8]. Thus, if phacovitrectomy was performed, according to the preoperative data, the mean 0.65-mm increase of LRD in our study leads to an approximate myopic shift of 1.63 diopters. This value is larger than that of others [1, 2, 7] in which patients who underwent silicone oil tamponade were excluded. Interestingly, age was positively correlated with △RLP and negatively with △LRD. Since the value of △RLP is negative, while that of △LRD is positive, these data synergistically indicate that the anterior shifting of the lens after silicone oil tamponade lessens in older patients. One possible explanation might be the thicker and denser nature of the lens in older individuals.

There are also limitations to the present study. Ocular biometric measurements in these phakic eyes after silicone oil removal should be analyzed in the future. Of note, postoperative myopic shift after phacovitrectomy also occurs in patients with gas tamponade, which is caused by the buoyancy and surface tension of the gas, persisting even after the gas has disappeared 1 month postoperatively [4]. Further studies are needed to observe the prolongation of LRD in eyes with phacovitrectomy and the final degree of myopic shift after silicone oil removal.

## Conclusion

In this study, we collected ocular biometric measurements using the IOLMaster 700 and found that not only underestimation of AL but also thickening and anterior shifting of the crystalline lens in phakic, macula-off RRD eyes that had undergone vitrectomy with silicone oil tamponade. These biometric changes contribute to a longer LRD, potentially resulting in the observed postoperative myopic shift. Our results should be interpreted with these limitations in mind. We did not measure the height of macular detachment, and new approaches are required to improve these measurements.

## Data Availability

The datasets used and/or analysed during the current study available from the corresponding author on reasonable request.
